# Determining the feasibility for an overdose prevention line to support substance users who use alone

**DOI:** 10.1186/s12954-022-00670-0

**Published:** 2022-08-19

**Authors:** Kim Ritchie, Sumantra Monty Ghosh

**Affiliations:** 1 Grenfell Ministries, Hamilton, ON Canada; 2 Internal Medicine, Disaster Medicine, and Addiction Medicine, Department of General Internal Medicine and Neurology, University of Alberta, Edmonton, Canada; 3 Department of Medicine and Psychiatry, University of Calgary, Calgary, Canada; 4 Rapid Access Addiction Medicine, 707 10 Ave SW 3rd Floor, Calgary, AB T2R 0B3 Canada

**Keywords:** Harm reduction, Opioid use disorder, Virtual supervised consumption, Overdose prevention, Remote monitoring

## Abstract

**Introduction:**

The majority of opioid-related deaths occur in suburban communities with people who use alone in their homes.

**Background:**

To reach individuals who use substances alone, Grenfell Ministries, a not for profit agency in Hamilton Ontario created a phone-based supervision service to target individuals who use substances alone.

**Methodology:**

Grenfell implemented a phone line service initially as a 3-month pilot eventually operationalized to a 24/7 phoneline to determine utilization of the service and test operational feasibility. Metrics such as timing of use, number of unique clients using the service and substances used were measured.

**Results:**

The line was provincially utilized. Between February 1st and December 10, 2020, the line was used 64 times. Most calls occurred in the evening, with fentanyl being the most used substance. EMS was dispatched 3 times for overdoses, of which 2 individuals were successfully resuscitated, and one individual’s status being unknown.

**Conclusion:**

The overdose prevention line can be implemented to support individuals who use alone. The service can successfully reduce risk of death from individuals who use alone and could be a valuable tool in addressing the opioid crisis. Further study needs to be conducted to determine its efficacy and safety in supporting clients who use alone.

## Key points/summary

- Individuals who use opioids alone make up the majority of overdoses in Canada.
- A telephone-based harm reduction service provides virtual support for individuals who use substances alone by remotely monitoring clients as they used substances like fentanyl.
- The telephone-based supervised consumption service was successfully able to prevent the death of two individuals who overdosed while using the service.
- A third individual who had overdosed had EMS services successfully dispatched in a timely manner, although the outcome of that individual is unknown.
- Phone-based virtual supervised consumption is a feasible option to support individuals who use opioids and other substances alone and could be a valuable tool in addressing the growing opioid crisis.

## Introduction

The opioid overdose crisis has become a focus internationally. In response, Canada has instituted a multi-faceted response including naloxone kit distribution, opioid replacement therapy (ORT), education, and supervised consumption sites (SCS). The fourth initiative demonstrated a reduction in the rates of mortality, the transmission of HIV and other communicable diseases and improved engagement with community-based health services. These positive results from SCSs are geographically limited to only 500 m surrounding the SCS site [1]. This limited geographic effect, combined with the reluctance of some people to have an SCS in their community due to public safety concerns and competing interests, results in limited access to SCS [2].

Media coverage of the crisis has focused on individuals in the downtown core, but individuals who live with opioid use disorder (OUD) have diverse socioeconomic status and social locations. Data from Hamilton, Ontario, demonstrate that 72% of opioid-related deaths were males with an average age of 36 years, and the vast majority of these individuals were using alone [3]. Similarly, suburban areas account for 83% of overdoses, and 70–76% of opioid-related deaths in Alberta, well outside the therapeutic distance of physical SCSs, with upwards of 67% of fentanyl overdoses were using in their place of residence [4]. Many SCSs do not run 24/7 due to costs and staffing concerns, with the average cost of a single SCS estimated at $2.9 million per year [5, 6]. The inability to access services at all hours has become a significant barrier as substance use frequency and time varies.

The primary aim of this project is to determine the practicality and utilization of a telephone-based overdose response line as an adjunct to physical SCSs to enhance access to harm reduction resources. Key concerns evaluated were the timing of the service, what substances were being used, and if there was user acceptance based on recurrent use of the service.

## Methods

This study abides by the Standards for Quality Improvement Reporting Excellence 2.0 (SQUIRE 2.0) reporting guidelines. Ethics exemption was obtained from The University of Alberta. Our group theorized that a telephone-based line was a necessary step in advancing harm reduction practices due to limitations in the therapeutic geographic effectiveness of physical SCSs, along with the stigma associated with SCSs. Additionally, some substances using communities were already providing telephone support to one another during the administration of substances in order to prevent the death of their community members, hinting that the intervention would be acceptable. The intervention is a phone service where individuals who are using substances alone can call a toll-free number and connect with an operator on the line. The operator will then virtually monitor the client as they consume their substance. The operator remains on the phone, checking up on the client. If the client becomes unresponsive, the operator will contact 911 dispatch to send Emergency Medical Services (EMS) to their location.

### Overview of the technology

People of lived experience operate the line through a smartphone-based application called “Line2” purchased by Grenfell. Line2 is a private service that provides a toll-free number with unlimited calling and messaging for Canada, operating on all Canadian telecommunications networks. The Line2 Application also has a call queue function that allows for a call to be forwarded to another device when the primary operator is currently on another call, allowing for two calls to be answered simultaneously by two operators at one time. Up to 10 simultaneous callers can be answered, provided there are enough operators. Additionally, calls can be run through on multiple personal devices, ensuring that no calls are missed if, for instance, an operator’s device was damaged, misplaced, lost access to the internet or the battery failed.

### Implementation

Due to multisystem impact, Grenfell Ministries contacted key stakeholders to establish awareness and feedback on their novel intervention including Hamilton’s Paramedic Service, Municipal Healthy and Safe Communities Department, and members of the substance using community. The Hamilton Ontario Paramedic Service relayed that additional steps would simplify their job. These included:

- Turn on a porch light
- Put away any pets
- Leave the door unlocked
- Hide substance paraphernalia which can be confiscated by police services.

Additional questions to assist paramedics when they arrived at the scene included:

- Whether the caller had a naloxone kit
- Allergies and other medical conditions.
- Any other individuals in the overdose space who could assist during an emergency

The municipality had two additional mental health crisis lines known as Help 24/7 and Crisis Outreach and Support Teams (COAST). Discussions with these agencies led to further delineation regarding the scope of practice for outreach phone lines. A decision was made that if operators encounter cases requiring additional mental health and addiction supports outside the scope of the operator, the line operator would transfer the client to these agencies to prevent the line from being tied up and reduced the amount of training the operators required.

Operators, who are all volunteers, were initially on boarded via phone interview and participated in online training which is approximately 4 h in length and consists of both video and written format training with a quiz assessment. The training curriculum is outlined in Table 1. A phone interview is then conducted to answer any questions and see if the individuals comprehend the concepts of harm reduction and the technical functions of the line. The operators managed the line from the comfort of their home using the supplied Line2 technology. The volunteer operators had various backgrounds and often worked in harm reduction and social services.

**Table 1:** Overview of the training curriculum for the operators of the overdose response line

Risk factors for opioid overdose	Loss of tolerance	
	Mixing drugs	
	Using alone	
	New dealer or new supply	
	Using with alcohol and benzodiazepines	
Actions	When to call 911?	
	When to refer to a crisis line and which would be most effective?	
	When to report a concern or overdose?	
Confidentiality	What confidentiality means?	
	What it means in relation to the Overdose Prevention Line?	
Acquire and confirming location	Using google street view to confirm location and why it is necessary	
	What questions to ask regarding location?	
Data collection	What data is needed to collect and why?	
	How to collect data?	
	Where to send data report at the end of shift?	
What we are and what we are not	Know our mission and purpose	
	What is out of our scope of practice?	
	What we are	What we are not
	A line of support for those who use drugs, main goal is to attempt to be able to dispatch emergency services should an overdose occur	A crisis line, a support line, a community connection service, counsellors, emergency responders, doctors, psychiatrists, or mental health specialists
Knowledge	Safe injection practices	
	Safe injection locations on the body	
	Up to date public health harm reduction information	
	Hours of operation of nearest safe injection site and location	
	List of national support lines	
	Where to get addiction treatment and opioid agonist therapy (OAT) if a client so desires?	
	Where to get mental health supports if required?	

Initially for the pilot, the line was only active 12–14 h a day, but on August 30th, 2020, the line went 24/7. The dynamics of the management team was adapted, and instead of having one phone line supervisor, six were put into place to cover the line for the full 24 h. The phone line supervisors offered peer support to operators and answer any calls missed by the operators. The overdose prevention line coordinator managed the team of phone line supervisors. Other positions include the program director, whose role entails cataloguing all the data received from calls and navigates any complaints or concerns brought up by staff or callers. There was also tech support, which assisted operators with any technical challenges operators encountered while using the line.

WhatsApp was used to facilitate communication between the various staff including the six phone line supervisors and the upper management team to discuss and develop weekly schedules and to build connection and rapport with communities across the province of Ontario including emergency providers. The WhatsApp application assisted in building team cohesion, increased operational efficiency and communication, and lastly aimed to reduce the potential adverse effects of self-isolation by providing both connection and purpose to our operators. The operators provided each other with peer-support through the chat and shared light conversation, funny memes, and pictures.

### Legal consideration

In 2016, The Canadian Federal Government established the Good Samaritan Drug Overdose Act. This Act provides some legal protection for individuals who seek help during an overdose emergency. This Act protects clients with possession of any controlled substances or protection of breach of specific conditions such as probation and parole secondary to substance possession. This Act was crucial to implementing this intervention, as it encourages clients to utilize the service without fear of repercussions secondary to possession. The Act, however, does not protect against those with pending warrants, substance production, or trafficking of illicit substances [7]. Legal advice was obtained regarding liability, which allowed Grenfell to start the line but placed most of the legal burden on the organization. Liability and director’s insurance was obtained to protect the organization, OPL operators, and volunteers of the line if poor outcomes occur.

### Barriers and Hurtles

The overdose prevention line pilot project afforded opportunities to explore the dynamics of engineering a new form of harm reduction. One of the first challenges posed was that of the limits of the technology itself.

If an operator’s internet or device battery failed, or if the device became damaged, the operator would not be able to access the application. A solution to reduce the possibility of a missed or dropped calls was to have both the phone line operator and phone line supervisor sign into the application at one time. Another more complex hurdle was that substance-using communities often distrust society in general due to years of stigmatization and discrimination experienced by people who use substances. As noted above, prior to the 2016 Drug Overdose Protection Act, those who sought out assistance or were experiencing an overdose could be charged with possession of a controlled substance. Before the line could be considered a safe and reliable resource in the drug-using community, trust would need to be earned. Public relations efforts such as the selfie campaign, seeking feedback from substance-using communities, as well as Grenfell Ministries’ firm public stance on social issues that directly impact these communities, allowed for the development of trust with these communities.

### Measurements and metrics for quality improvement

As this service was one of the first of its kind, initial metrics were gathered with the view to examine impact by determine the following: Who was using the line, when were they using the line, what were they using the line for, and what were the outcomes of using the service. The service started with limited hours of operation; thus, it was prudent to determine when the service was most utilized to help determine how best to concentrate services. This included looking at the number of phone calls, purpose of the calls, and determining when the service was most utilized by examining the timings of phone calls. In regards to unique caller identification, similar measurement strategies to the Ontario supervised consumption services were used through a unique coding system. To gain an understanding if there was some predictability to overdoses or other adverse outcomes on the line, we measured the type of substance being used, method and route of use, and the amount. Also essential to the service were the addresses of the callers to help the operators know where to send the Emergency Medical Service (EMS) team. For data collection purposes and to protect the privacy of the clients, only the city was documented in the records. All data metrics are summarized in Table 2.

**Table 2:** Information and measurements recorded for the phone line

Measurement indicator	Purpose
Phone call ID using first two initials of first name, first two initials of last name, and last two digits of year of birth	To identify unique callers
Location of call	To determine where to send EMS responses
Time of phone call	To determine when call volumes were highest
Purpose of phone call	To determine if the calls were for substance use or other reasons such as learning about the line
Self-reported substances used	To determine which substances were most used during for the line and its potential impact on how quickly someone may overdose
Self-reported route of use	To determine the potential impact of how quickly someone may overdose
Self-reported quantity of substance used	To determine the potential impact of how quickly someone may overdose

## Results

The evaluation of the pilot program began with the commencement of the service on February 1, 2020, to May 16, 2020. In total, the line was called 14 times by 8 unique users who used substances, and 68 of the calls were from service providers wanting to know more about the service being provided. Of all the service calls, EMS services were only dispatched once. After the pilot phase had ended, between May 17 and August 30th over 15 calls were conducted with no EMS dispatches with 5 unique users of substances. After August 31st, 35 phone calls were conducted with 7 unique callers. Overall, three overdoses occurred with three unique individuals in three different cities in Ontario. EMS services were called via 911 dispatch in Hamilton Ontario, two of which were patched through to the two other cities where these overdoses occurred. Of these three, two were successfully reversed as determined by call backs from these clients who continued to use the service, and one outcome was unknown as the call was discontinued when EMS services arrived on scene. All three overdoses were attributed to fentanyl use.

Clients had called from multiple jurisdictions in Ontario, some of which are displayed in Table 3. Uniquely, the Hamilton 911 dispatch service can connect with all other dispatch services in the province allowing Grenfell to support clients province wide.

**Table 3:** Phone call locations in Ontario during the pilot phase of the study

Ontario cities service users called from	Number of unique callers
Cornwall	1
Guelph	4
Hamilton	3
London	1
St. Catherine’s	1
Not disclosed	4

From the initial pilot phase of the non-service utilization calls (i.e., individuals who did not use the service to support substance use), 72% were service providers wanting more information about the service for their clients. Three percent were from individuals who were supporting the individuals using the service. Nine percent were question calls, 9% were wrong numbers, 3% were missed calls with no left-over numbers, and 4% were off-hour calls with no response.

### Types of substances used and service utilization

**Fig. 1:**
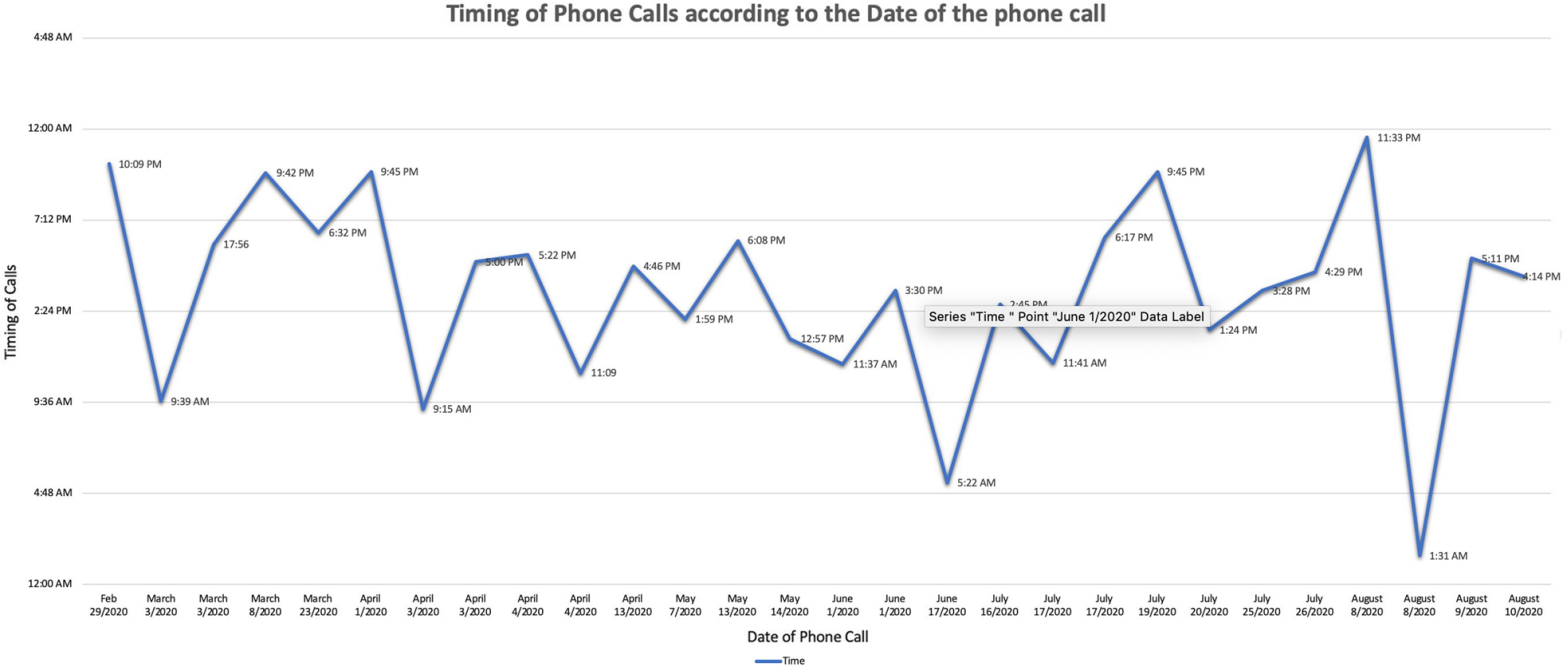
Timing of phone calls over a 6-month period of the overdose prevention line service

For the pilot phase, nine out of the 14 calls used injection forms of substance consumption. The typical range of fentanyl used was 0.75–1.5 points. Dilaudid was also utilized at 10 mg, and morphine was used at 30 mg tablets. Figure 1 displays the percentages of substances utilized during the calls collectively. After May 19, 14 more calls for service provision were conducted, with 12 of the 14 calls related to fentanyl use, and two related to stimulants. Five unique callers were found to use the service consistently over the months.

### Plan design study act

Due to limited staffing support, knowledge on service utilization and ways to make the program more efficient was required. Plan, Do, Study, and Act (PDSA) cycles were initiated.

Initially, the group utilized volunteers who performed 12-h shifts to provide comprehensive service provision. Data was collected by the operator by logging into a private membership portal through Grenfell Ministries. The data was used to determine how best to structure the service provision timing, including shift changes and when more staff would be required.

**Fig. 2:**
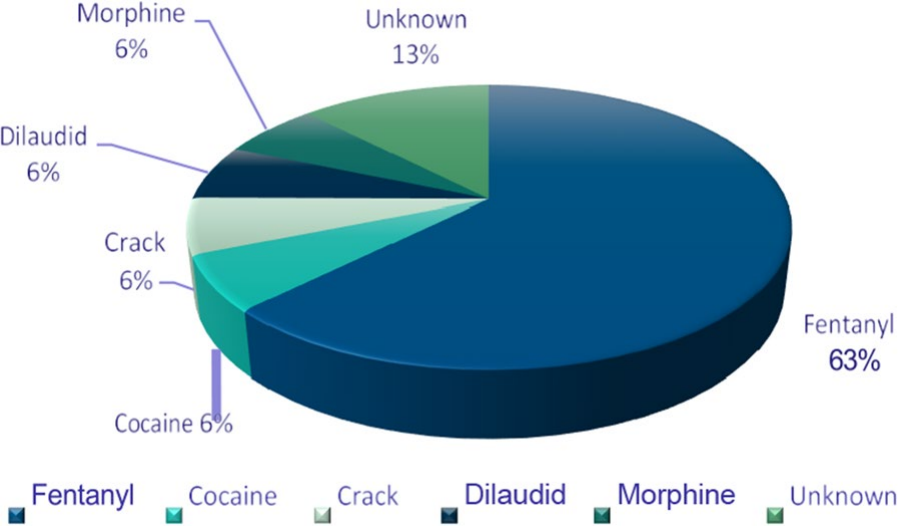
Percentages of substances being used during the pilot phase

Using the number of calls where actual service was provided, a run diagram examining the frequency and timings of calls was constructed to predict how to concentrate services. Balance measures of missed calls were also used. The timing of phone calls was significantly concentrated toward the afternoons and evenings (Fig. 2). This is reflective of many substance use patterns being focused toward the evening. While the line was not open after midnight, clients still could leave messages and the operators of the line to know who called the phone line and when. There was limited calling of the line after hours.

Grenfell Ministries committed itself to provide extended hours of service coverage during the first 6 weeks of the operation of the line to gather data on the time of the highest call volume. After 6 weeks, it was noted that most calls occur between the afternoon and evening. The hours were then switched from the original Monday to Friday from 10:00 AM–10:00 PM and Saturday and Sunday from 10:00 pm-midnight to Monday to Friday from 12:00 PM–10:00 PM and Saturday and Sunday from 12:00 PM–12:00 AM.

Within 5 months of the OPL launch, changes were made to run the line 24/7. To accommodate such an undertaking, the line was saturated with phone line operators during peak times; the operators were allowed to sign in and out of shifts whenever they want to, for as long or as short as the operator needs the shift to be. Additionally, a crew of six phone line supervisors was created. They had strict scheduled shift times to catch any calls that may not be answered by the operators. The supervisors will also offer debriefing services, peer counseling, answering questions and concerns to all the operators. An overdose prevention line coordinator managed the supervisor team and negotiated schedule changes, assisting supervisors while they are logged into the line such as debriefing and peer support. Using WhatsApp chat, the phone line supervisors inform everyone they have logged into their shifts enabling the volunteer operators to know whom to seek support from during their shift.

Of note, during the initial pilot, some calls were missed due to only one operator being online at a time. The Line 2 phone application used as the backbone of the phoneline had some limitations, including concerns regarding phone data or WiFi becoming unavailable limiting operator access to the application. A design strategy was created where both the phone line operator and the supervisor signed into the application simultaneously and were both available for clients, thereby reducing the chances of a call being missed.

## Discussion

To our knowledge, this is the first evaluation of a phone-based overdose prevention line. The overdose prevention line provides increased access to harm reduction services to individuals who use alone. While the data is limited, the intervention of using a virtual supervised consumption service in the form of an overdose response line had demonstrated some acceptability for use as well as feasibility, and lastly efficacy in reducing overdose risk. With 9 months’ worth of data, the phone line has demonstrated reasonable geographic penetration with calls from clients from all over Ontario, although the number of calls was overall limited. Clients were effectively observed, and three call out was made for an overdose, two of which were saved upon administration of naloxone. Feedback obtained from operators suggested reasonableness in working with clients, and there were clients who returned to use the service, suggesting some acceptability of the intervention. Operators felt the substance, route, and quantity reported by the users were helpful in determining how quickly someone could potentially overdose.

While the project is being promoted, user uptake has been limited but steadily expanding. Limitations of the service are proportional to demand of the service from service users. As the demand for the service was limited, volunteers were able to keep up with the demand for calls, but this may change if volume of demand increases. Of note, the service was terminated on December 10, 2020 to and pivoted to the National Overdose Response Service which launched nationally on December 15, 2020. This full-scale service is currently being evaluated from an implementation science framework using mixed methods analysis. Operator to client ratios will need to be examined in future iterations of the project to ensure appropriate scaling.

Some key limitations in the generalizability of the work are that Ontario has a provincial 911 dispatch system, which makes the service accessible provincially. This is not necessarily the case with other jurisdictions. The legal aspects of the service have not been fully delineated and proven to be a bottleneck for other jurisdictions creating this service. Discussions with stakeholders highlighted further concerns with clients worried about concurrent police dispatches with EMS, posing risk to substance users who were adverse to interacting with police, especially those who are racialized. There were concerns that some overdoses are quite rapid, and EMS services may not arrive on time to resuscitate an overdose victim.

Further research on the clinical effectiveness of the service need to be determined. Efforts to understand its clinical effectiveness have been proposed in the Canadian province of Alberta with a newly published study protocol to assess implementation outcomes [8]. This protocol will help support the NORS evaluation over the coming year.

## Conclusion

In this quality improvement study, a phone-based overdose prevention line was used to reach individuals outside the therapeutic distance of an SCS. This intervention may be impactful to the current opioid crises providing support for substance users who use alone.

## Data Availability

Data can be shared with third parties if a reasonable request is made. Data from our study can be made available upon request.
